# Supplementary material for: The Correlates & Public Health Consequences of Prospective Vaccine Hesitancy among Individuals Who Received COVID-19 Vaccine Boosters in the U.S
Source: Vaccines (Basel). 2022 Oct 25;10(11):1791. doi: 10.3390/vaccines10111791 (PMC9698316; doi:10.3390/vaccines10111791)
Supplement: Supplementary file 1 [file vaccines-10-01791-s001.zip › vaccines-1964333-supplementary.pdf]

Supplemental Materials For: The Social Correlates & Health Policy Consequences of Prospective Vaccine Hesitancy Among Individuals who ReceivedCOVID-19 Vaccine Boosters.

Contents

Table S1. Comparison of Raw and Weighted Lucid Data to National Benchmarks 2

Supplemental Measurement Information 3

COVID-19 Vaccine Uptake (Initial Series) 3
COVID-19 Vaccine Booster Status 3
Additional Booster Intentions 3
Political Ideology 3
Additional Demographics 4

Table S1. Comparison of Raw and Weighted Lucid Data to National Benchmarks

Variable	Our Data (Raw)	Our Data (Weighted)	Benchmark	Benchmark Source
Female	52%	51%	51%	CPS 2018
College Degree	39%	32%	31%	CPS 2018
Black	10%	13%	13%	CPS 2018
White	69%	63%	62%	CPS 2018
Hispanic	12%	17%	18%	CPS 2018
Democrat	34%	34%	34%	ANES (Wgt.)
Republican	24%	24%	28%	ANES (Wgt.)
Independent	42%	40%	32%	ANES (Wgt.)
Mean Age	45	46	47	ANES (Wgt.)
Median Income	$ 45 - 49,000	$ 60 - 64,999	$ 55-59,999	ANES (Wgt.)

Note. Comparison of our data to known population benchmarks. CPS = Current Population Survey (US Census, 2018). ANES = American National Election Study (2016). We prefer to rely on CPS given its sample size and representativeness, but make use of weighted ANES data whenever it was not possible to use CPS (e.g., CPS does not ask questions about Party ID). Weights in column two adjust for gender, education, race, age, and income. Party ID is **not included** in our weighting formula, and is shown only due to the potential interests of those who might use or otherwise consume this data.

Supplemental Measurement Information

COVID-19 Vaccine Uptake (Initial Series)

Have you been fully vaccinated against COVID-19?
(E.g., Two doses of Pfizer or Moderna;
1 dose of Johnson & Johnson)

ROTATE RESPONSE OPTION ORDER

<1> Yes
<2> No

COVID-19 Vaccine Booster Status

IF COVID VACCINE STATUS = YES

Have you received an additional "booster"
COVID-19 vaccine in the past year?

<1> Yes
<2> No -- but I plan to
<3> No -- and I do not plan to

Additional Booster Intentions

IF BOOSTER STATUS = YES

As you may know, public health experts now recommend that all adults receive an additional "booster" dose of the COVID-19 vaccine. They have also authorized additional (second) boosters for vulnerable populations.

If, in the future, federal regulators were to recommend that all Americans receive an additional (second) booster shot, in order to reduce the likelihood of becoming sick with COVID-19 and/or spreading it to others, how likely would you be to take the shot?

<1> Very likely
<2> Somewhat likely
<3> Not too likely
<4> Not likely at all
<5> I have already received a second booster for COVID-19

Political Ideology

We hear a lot of talk these days about liberals and conservatives. Here is a seven-point scale on which the political views that people might hold are arranged from extremely liberal to extremely conservative. Where would you place yourself on this scale?

<1> Extremely liberal
<2> Liberal

<3> Slightly liberal
<4> Moderate; middle of the road
<5> Slightly conservative
<6> Conservative
<7> Extremely conservative

Additional Demographics

All other demographic variables are measured as part of Lucid’ s initial inventory survey, when inviting prospective panelists to join the service’ s large, online, opt-in panel. Additional information about these measures can be found in the main text.
